# Characterization of the metabolic effect of β-alanine on markers of oxidative metabolism and mitochondrial biogenesis in skeletal muscle

**DOI:** 10.20463/jenb.2016.06.20.2.5

**Published:** 2016-06-30

**Authors:** Jamie K. Schnuck, Kyle L. Sunderland, Matthew R. Kuennen, Roger A. Vaughan

**Affiliations:** 1 Department of Exercise Science, High Point University, High Point North Carolina U.S.A.

**Keywords:** Carnosine, peroxisome proliferator-activated receptor γ coactivator 1α (PGC-1α), peroxisome proliferator-activated receptor β/δ (PPARβ/δ), glucose transporter 4 (GLUT4)

## Abstract

**[Purpose]:**

β-alanine is a common component of numerous sports supplements purported to improve athletic performance through enhanced carnosine biosynthesis and related intracellular buffering. To date, the effects of β-alanine on oxidative metabolism remain largely unexplored. This work investigated the effects of β-alanine on the expression of proteins which regulate cellular energetics.

**[Methods]:**

C2C12 myocytes were cultured and differentiated under standard conditions followed by treatment with either β-alanine or isonitrogenous non-metabolizable control D-alanine at 800μM for 24 hours. Metabolic gene and protein expression were quantified by qRT-PCR and immunoblotting, respectively. Glucose uptake and oxygen consumption were measured via fluorescence using commercially available kits.

**[Results]:**

β-alanine-treated myotubes displayed significantly elevated markers of improved oxidative metabolism including elevated peroxisome proliferator-activated receptor β/δ (PPARβ/δ) and mitochondrial transcription factor a (TFAM) which led to increased mitochondrial content (evidenced by concurrent increases in cytochrome c content). Additionally, β-alanine-treated cells exhibited significantly increased oxygen consumption compared to control in a PPARβ/δ-dependent manner. β-alanine significantly enhanced expression of myocyte enhancer factor 2 (MEF-2) leading to increased glucose transporter 4 (GLUT4) content.

**[Conclusion]:**

β-alanine appears to increase cellular oxygen consumption as well as the expression of several cellular proteins associated with improved oxidative metabolism, suggesting β-alanine supplementation may provide additional metabolic benefit (although these observations require in vivo experimental verification).

## INTRODUCTION

**Figure 1.**
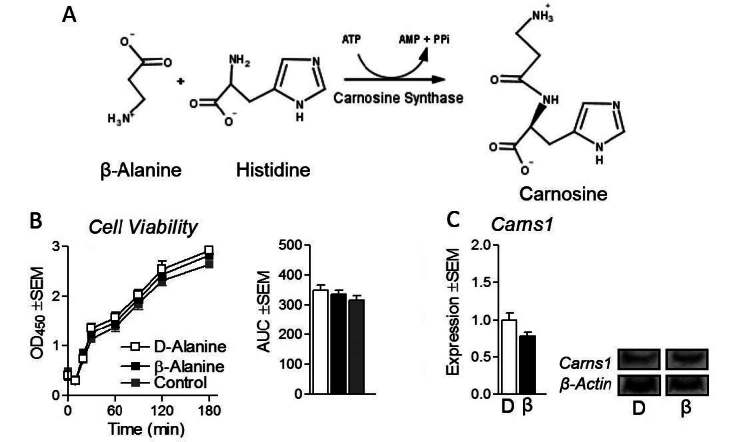
Carnosine biosynthesis, Carns1 expression, and cell viability. (a) Chemical reaction of carnosine biosynthesis mediated by carnosine synthase 1 (Carns1). (b) Cell viability following treatment of C2C12 myotubes with isonitrogenous and non-metabolizable D-alanine (D) at 800μM, or β-Alanine (β) at 800μM or true control (media) for 24 hours. (c) Protein expression of Carns1 following treatment as described above. NOTES: Target protein expression was normalized to β–actin protein expression. Chemical reaction of carnosine synthase was drawn using ChemAxon MarvinSketch available from: http://www.chemaxon.com/marvin/

β-alanine (3-aminopropanoic acid) is a non-essential amino acid found in many commercially available over the counter sports supplements that are sold to enhance athletic performance and prolong exercise duration^2,3,18^. β-alanine functions (at least in part) by combining with the conditionally essential amino acid histidine to generate carnosine by the enzyme carnosine synthase (Carns1) (Fig 1a), thereby functioning as an intracellular buffer^14,18,23^. During exercise, continuous myosin-actin crossbridge formation leads to increased ATP hydrolysis and subsequent proton formation thereby contributing to reduced pH and possibly acidosis^22^. Supplementation with β-alanine has been shown to enhance muscle carnosine synthesis and content in a dose-dependent manner^10, 15^, and delay accumulation of lactate during exercise through buffering the formation of lactate from pyruvate^18^. β-alanine supplementation is commonly used for anaerobic exercise due to the proton buffering capability of carnosine^14, 18, 23^. Evidence demonstrates a moderately beneficial effect of β-alanine supplementation for exercise with a substantial contribution from oxidative metabolism^11, 26, 27^. β-alanine supplementation has been shown to increase time to exhaustion (TTE) in exercise lasting ~20 minutes with^26^ and without^27^ concurrent exercise training. Specifically, Smith et al. demonstrated a significant increase in VO2 peak, TTE, and total work completed in healthy male subjects supplemented with β-alanine for 6 weeks compared with un-supplemented controls, both of which received concurrent exercise training^26^. Remarkably, female subjects supplemented with 6.4g/day for the majority of the 28 day trial also exhibited increases in TTE, suggesting β-alanine may provide benefits similar to exercise training^27^. And while β-alanine is generally considered safe^2, 14, 16, 23, 24^, some side effects such as skin irritation or burning sensation (otherwise known as paraesthesia) can occur^23^, leading some to caution the use of β-alanine until more is known about its safety^21^. Multiple reviews exist regarding the efficacy and potential effects of β-alanine supplementation for improving athletic performance, however to the best of our knowledge, and despite the observations described above demonstrating β-alanine’s ability to improve aerobic performance^11, 26, 27^, there have been no reports investigating the effect of β-alanine on indicators of oxidative metabolism in skeletal muscle^4, 21,24^. This report uniquely describes the effect of β-alanine on metabolic gene and protein expression associated with oxidative metabolism in cultured skeletal muscle.

Specifically, this work investigated the effects of 24 hour β-alanine treatment on markers of mitochondrial biosynthesis using the highest physiologically documented concentrations of β-alanine in human subjects (approximately 800μM)15. Peroxisome proliferator-activated receptor γ coactivator 1α (PGC-1α), a transcriptional coactivator that is considered the master regulator of mitochondrial biosynthesis^19, 31^, as well as downstream targets nuclear respiratory factors 1 (NRF1) and mitochondrial transcription factor A (TFAM) were examined^5, 25, 30^. NRF1 acts to promote mitochondrial biosynthesis through induction of TFAM leading to mtDNA replication and mtRNA expression^12, 13^. In addition to PGC-1α expression, we also investigated the expressional response of peroxisome proliferator-activated receptor α (PPARα) and peroxisome proliferator-activated receptor β/δ (PPARβ/δ) in β-alanine-treated cells. PPARs act as nuclear receptors which regulate fatty acid oxidation and transcription of respiratory chain components in metabolically active tissues (liver and skeletal muscle), with greatest emphasis placed on PPARβ/δ the predominant regulator of fatty acid metabolism in skeletal muscle^7, 20^. Additionally, we measured oxygen consumption as an indicator of oxidative metabolism with and without the presence of selective PPARβ/δ inhibition. Lastly, we investigated the expressional profile of numerous cytosolic proteins responsible for regulating the PGC-1α/PPAR axis, to further assess the mechanism(s) by which β-alanine may act to alter skeletal muscle oxidative phenotype.

## METHODS

### Cell culture

C2C12 mouse myoblast from ATCC (Manassas, VA) were cultured in Dulbecco’s Modified Eagle’s Medium (DMEM) containing 4500mg/L glucose and supplemented with 10 % heat-inactivated fetal bovine serum (FBS) and 100U/mL penicillin/streptomycin, in a humidified 5% CO2 atmosphere at 37°C. Cells were seeded overnight and grown to confluence with growth media changed every two days. Differentiation was accomplished by replacing growth media with DMEM supplemented with 2% horse serum and 100U/mL penicillin/streptomycin for 6 days. Stock β-alanine and D-alanine (serving as an iso-osmolar, isonitrogenous control) from Sigma (St. Louis, MO) were dissolved in culture media to a final concentration of 800 µM. This concentration was used because to our knowledge, 800µM is the highest documented plasma concentration of β-alanine achieved following supplementation in healthy volunteers^15^.

### Cell viability

Cells were treated with either a true media control or as described above for 24 hours. Following treatment, differentiation media, was replaced with differentiation media containing 10% WST (v/v). Colorimetric signal intensity was measured at 450nm temporally for 3 hours. Cell viability was not altered by either D-alanine or β-alanine treatments versus each other or true control (Fig 1b).

### Quantitative real time polymerase chain reaction (qRT-PCR)

Following differentiation and treatment as described above, total RNA was extracted using the Trisol method and cDNA was synthesized using the iScript cDNA Synthesis Kit from Bio-Rad (Hercules, CA) according to manufacturer’s instructions. PCR primers were designed using Primer Express software from Invitrogen (Carlsbad, CA) and synthesized by Integrated DNA Technologies (Coralville, IA). Amplification of target genes was normalized to the housekeeping gene, TATA Binding Protein (TBP). Table 1 summarizes the forward and reverse primers of each gene. qRT-PCR reactions were performed in triplicate using the CFX Connect System from Bio-Rad (Hercules, CA). SYBR Green based PCR was performed using six replicates per treatment with final primer concentrations at 10µM in a total volume of 20 µl using 100ng of sample cDNA per well (determined via NanoDrop from Thermo Fisher, Wilmington, DE). The following cycling parameters were used: 95˚C for 3 minutes followed by 40 cycles of 95˚C for 15 seconds, and 60˚C for 30 seconds. qRT-PCR reactions were performed in duplicate using lysates from 6 replicate wells per experiment. Relative quantification was determined via ΔΔCt method.

**Table 1.** Summary of qRT-PCR primers from Integrated DNA Technologies (Coralville, IA). Abbreviations: Carnitine palmitoyl tranferase 1b (CPT1b), forkhead box protein 1 (Foxo1), forkhead box protein 3 (Foxo3), lactate dehydrogenase a (LDHa), lactate dehydrogenase b (LDHb), myocyte enhancer factor 2 (MEF-2), nuclear respiratory factor 1 (NRF1), peroxisome proliferator-activated receptor γ coactivator 1α (PGC-1α), NAD+-dependent deacetylase sirtuin 1 (SIRT1), NAD+-dependent deacetylase sirtuin 3 (SIRT3), TATA binding protein (TBP), and mitochondrial transcription factor A (TFAM).

Primer Name	Forward Sequence	Reverse Sequence
CPT1b	5’-GTGACTGGTGGGAAGAATATGT-3’	5’-GGGTAAGAACTGGAAGCAGTAG-3’
Foxo1	5’-GTACAGACAGTGGCAGGATTAG-3’	5’-GATGGACGGAATGAGAGGTAAA-3’
Foxo3	5’-CTGAAGGATCACTGAGGAAAGG-3’	5’-CTGCAGGTTACTGTGTGTAGAA-3’
LDHa	5’-GGCTTGTGCCATCAGTATCT-3’	5’-CCCGCCTAAGGTTCTTCATTAT-3’
LDHb	5’-GAACTGGAAGGAGGTGCATAA-3’	5’-GCTCCTAGTGCAAACATCAAAC-3’
MEF-2	5’-CAAACCCTCGACACGATTCT-3’	5’-ACGGTGTGTGTGCCTAATAC-3’
NRF1	5’-ACCCTCAGTCTCACGACTAT-3’	5’-GAACACTCCTCAGACCCTTAAC-3’
PGC-1α	5’-GACAATCCCGAAGACACTACAG-3’	5’-AGAGAGGAGAGAGAGAGAGAGA-3’
SIRT1	5’-ACCGATGGACTCCTCACTAA-3’	5’-ATCTGCCACAGCGTCATATC-3’
SIRT3	5’-GGAGGAAGCAGTGAGAAGAAG-3’	5’-CCCGTCGATGTTCTGTGTATAG-3’
TBP	5’-GGGATTCAGGAAGACCACATA-3’	5’-CCTCACCAACTGTACCATCAG-3’
TFAM	5’-GAAGGGAATGGGAAAGGTAGAG-3’	5’-ACAGGACATGGAAAGCAGATTA-3’

### Immunoblotting and protein expression

Following differentiation and treatment of 6 replicates per treatment, whole cell lysates were prepared by harvesting the cells on ice in Ripa buffer from Bio-Rad (Hercules, CA) supplemented with protease inhibitor mix from Bio-Rad (Hercules, CA), followed by incubation on ice for 60 minutes. Protein concentrations were determined by Bradford assay from Bio-Rad (Hercules, CA). Total protein (50 μg per sample) of treatment-pooled lysates was size-separated in triplicate by 10% sodium dodecyl sulfate polyacrylamide gel electrophoresis (SDS-PAGE) and electro-transferred to PVDF membranes. After blocking in TBST-5% non-fat milk powder for 1 hour, membranes were probed at 4ºC for overnight with primary antibodies for each target as well as anti-β-actin primary monoclonal antibody from Santa Cruz Biotechnologies (Santa Cruz, CA) in TBST-5% non-fat milk powder overnight. Bound antibodies were detected by horseradish peroxidase-conjugated secondary antibodies from Santa Cruz Biotechnologies (Santa Cruz, CA) and by chemiluminescence using the Clarity Western ECL substrate kit from Bio-Rad (Hercules, CA) and imaged using the ChemiDoc Touch from Bio-Rad (Hercules, CA). Target proteins from western blots were quantified via densitometry and normalized to β-actin using Image Lab software from Bio-Rad (Hercules, CA).

### Glucose uptake

Glucose uptake was measured using a commercially available kit from Cayman Chemical (Ann Harbor, MI). Briefly, cells were glucose deprived for 2 hours, followed by treatment with glucose-free PBS containing the fluorescently-tagged glucose derivative 2-(N-(7-nitrobenz-2-oxa-1,3-diazol-4-yl)amino)-2-deoxyglucose (2-NBDG) at 200 μg/ml and incubated at 37˚C per manufacture’s recommendations. PBS containing 2-NBDG was then removed, rinsed with assay buffer, and fluorescent signal recorded using excitation/emission at 485/535nm.

### Oxygen consumption

Oxygen consumption was measured using a commercially available kit from Cayman Chemical (Ann Harbor, MI). Differentiation media containing each treatment was replaced with PBS containing glucose at 4.5 g/L with MitoXpress and individual wells were sealed with mineral oil per manufacture’s recommendations. Fluorescent signal was measured temporally for 4 hours using excitation/emission at 380/650nm.

### Statistical analyses

All data are presented as group means ± SEM. Immunoblots were analyzed by student’s t-test with significance determined by p < 0.05. Glucose uptake and oxygen consumption were analyzed via two-way ANOVA with Bonferonni’s correction for multiple comparisons.

## RESULTS

### Β-alanine stimulates mitochondrial biogenesis and related gene expression

**Figure 2.**
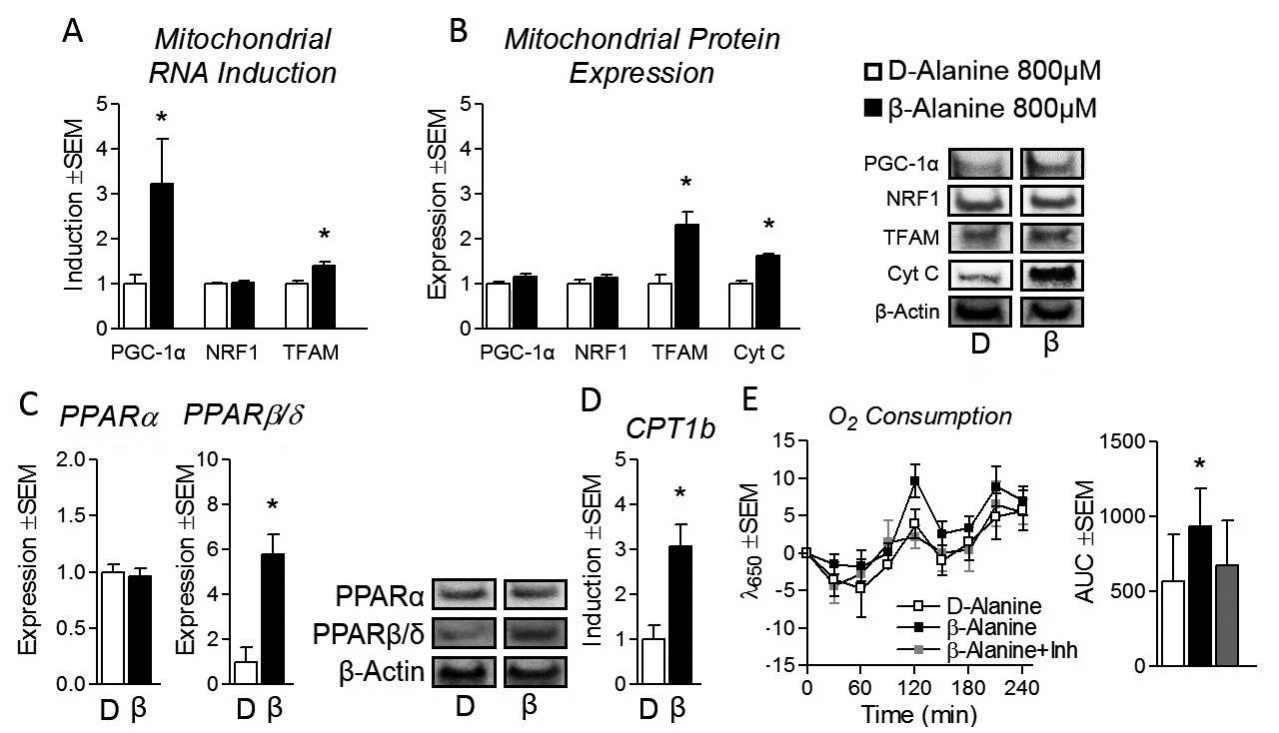
β-Alanine stimulates markers of mitochondrial biogenesis and oxidative metabolism. (a) Peroxisome proliferator activator receptor coactivator 1α (PGC-1α), nuclear respiratory factor 1 (NRF1), and mitochondrial transcription factor a (TFAM) mRNA expression of C2C12 myotubes treated with isonitrogenous and non-metabolizable D-alanine (D) at 800μM or β-Alanine (β) at 800μM or for 24 hours. (b) PGC-1α, NRF1, TFAM, and cytochrome c (Cyt C) protein expression following treatment as described above. (c) Protein expression of peroxisome proliferator-activated receptor α and β/δ (PPARα and PPARβ/δ) following treatment as described above. (d) RNA expression of carnitine palmitoyl transferase 1b (CPT1b) following treatment of C2C12 myotubes as described above. (e) Oxygen consumption following treatment described above with β-Alanine treated cells with and without the selective PPARβ/δ inhibitor GSK3787 (GSK) with time trial (left) and area-under-the-curve (AUC) (right). NOTES: Target protein expression was normalized to β–actin protein expression. Target gene expression was normalized to tata binding protein expression (TBP). *indicates p < 0.05

In order to assess the effects of β-alanine on myotube metabolism, we began by measuring carnosine synthase expression (catalytic enzyme for carnosine biosynthesis from β-alanine and histidine, summarized in Fig 1a). Carns1 expression was unchanged between the two treatments (Fig 1c). Next, to assess the effect of β-alanine on mitochondrial biogenesis, we measured the expressional profile of regulatory genes in this process. Master regulator PGC-1α RNA was significantly increased in β-alanine-treated myotubes compared with D-alanine-treated cells, as was TFAM expression (Fig 2a). In order to verify these findings, we measured protein expression of the aforementioned genes and observed significantly elevated TFAM content compared with D-alanine control (Fig 2b). To determine if heightened TFAM expression led to increased mitochondrial content, we measured cytochrome c (Cyt C) expression. β-alanine-treated cells displayed significantly elevated Cyt C expression compared with D-alanine control cells confirming increased mitochondrial content (Fig 2b). Next, we sought to measure the effects of β-alanine treatment on the expression of nuclear receptors PPARα and PPARβ/δ. Interestingly, PPARβ/δ but not PPARα protein expression was significantly increased 5.8 ±2.2 fold in β-alanine-treated cells (Fig 2c). Not surprisingly, mRNA expression of the PPARβ/δ downstream target carnitine palmitoyl transferase 1b (responsible for mitochondrial lipid transport) was also elevated in β-alanine-treated cells (Fig 2d). Next, to determine if β-alanine-induced PPARβ/δ expression promoted mitochondrial metabolism, we measured oxygen consumption following treatment as previously described with β-alanine-treated cells both with and without the selective PPARβ/δ inhibitor GSK3787. Interestingly, β-alanine-treated cells exhibited significantly elevated oxygen consumption compared with D-alanine controls which was abolished by concurrent PPARβ/δ inhibition (Fig 2e).

### Β-alanine stimulates markers of glycolytic metabolism

**Figure 3.**
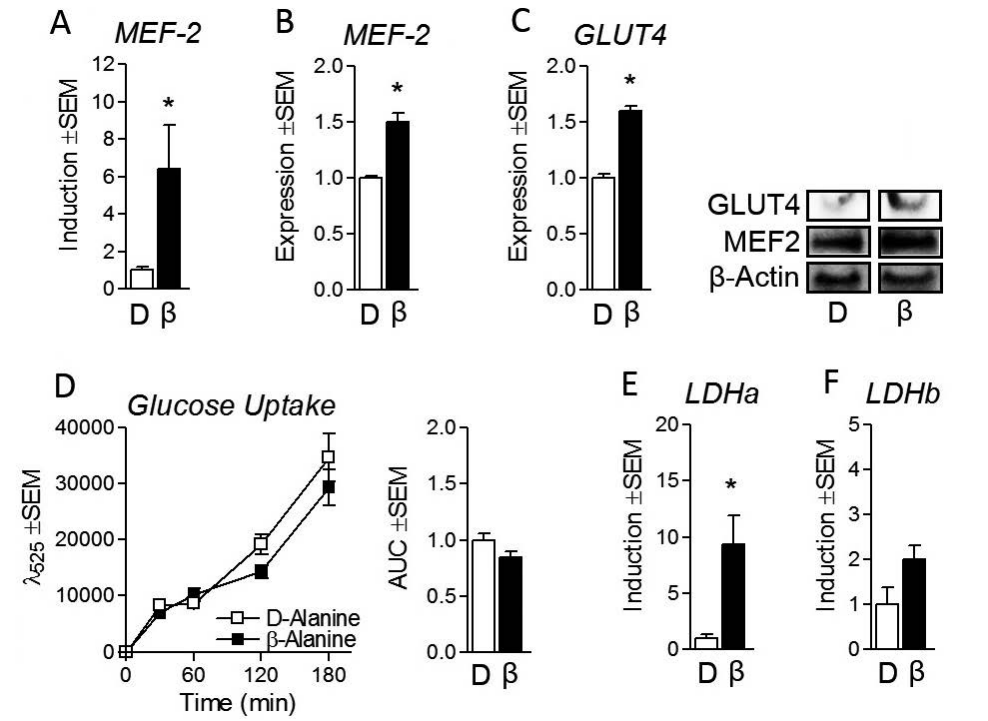
β-Alanine stimulates glucose transporter 4 via MEF-2. (a) Myocyte enhancer factor-2 (MEF-2) RNA expression following treatment of C2C12 myotubes with β-Alanine (β) at 800μM or isonitrogenous and non-metabolizable D-alanine (D) at 800μM for 24 hours. (b) Protein expression of MEF-2 following treatment of C2C12 myotubes as described above. (c) Protein expression of glucose transporter 4 (GLUT4) following treatment of C2C12 myotubes as described above. (d) Glucose uptake following treatment as described above with time trial (left) and area-under-the-curve (AUC) (right). (e and f) mRNA expression of lactate dehydrogenase A and B (LDHa and LDHb, respectively) following treatment as described above. NOTES: Target gene expression was normalized to tata binding protein expression (TBP). Target protein expression was normalized to β–actin protein expression. *indicates p < 0.05

Because cells were cultured in high-glucose media, we investigated the effects of β-alanine on indicators of glycolytic metabolism. β-alanine treatment resulted in elevated expression of myocyte enhancer factor 2 (MEF-2) at both the RNA and protein levels (Fig 3a and 3b, respectively). Additionally, the downstream target of MEF-2 (glucose transporter 4 or GLUT4) was also significantly elevated at the protein level (Fig 3c). To further investigate the effect of β-alanine on indicators of glucose metabolism, we measured glucose uptake which was not altered following β-alanine treatment compared with D-alanine control cells (Fig 3d). Interestingly despite no change in glucose uptake, we observed a significant induction of RNA expression in lactate dehydrogenase a (LDHa) but not lactate dehydrogenase b (LDHb), suggesting an increasing preference for lactate biosynthesis (Fig 3e and 3f).

### Cytosolic regulators of energy expenditure are unaffected by β-alanine

**Figure 4.**
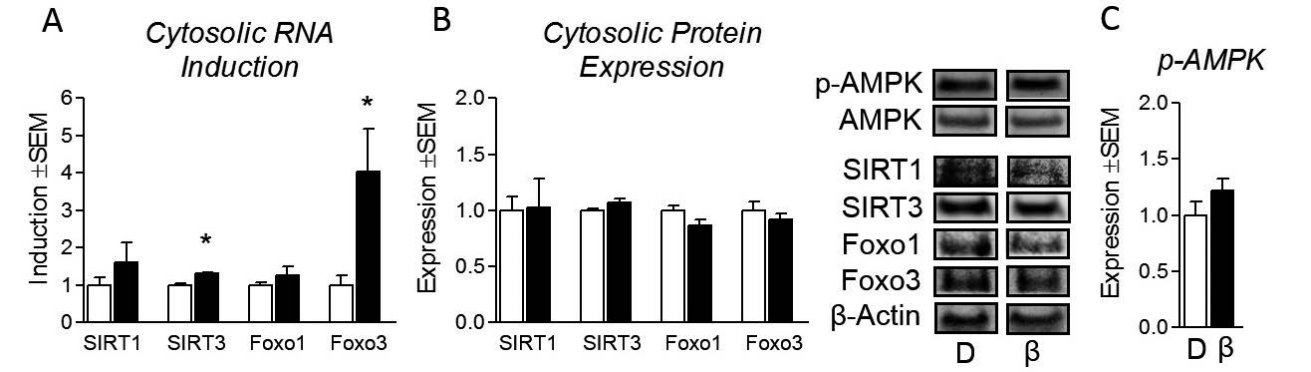
β-Alanine and expression of cytosolic metabolic regulators. (a) mRNA expression of NAD+-dependent histone deacetylase sirtuin 1 and 3 (SIRT1 and SIRT3) and forkhead box protein 1 and 3 (Foxo1 and Foxo3) following treatment of C2C12 myotubes with β-Alanine (β) at 800μM or isonitrogenous and non-metabolizable D-alanine (D) at 800μM for 24 hours. (b) Protein expression of the aforementioned genes following treatment as described above. (c) Protein expression of phospho-5’ AMP-activated protein kinase (p-AMPK) following treatment as described above. NOTES: Target gene expression was normalized to tata binding protein expression (TBP). Target protein expression was normalized to β–actin protein expression. *indicates p < 0.05

In order to elucidate some potential mechanisms of β-alanine-induced metabolic gene expression, we measured the expression of several cytosolic regulators of metabolism. We began by measuring the RNA expression of NAD+–dependent histone deacetylase sirtuin 1 and 3 (SIRT1) as well as forkhead box protein 1/3 (Foxo1/3) mRNA expression. Subtle but significant increases in SIRT3 and Foxo3 expression was observed following β-alanine treatment (Fig 4a). Despite elevated mRNA transcripts, protein expression of SIRT and Foxo proteins were unaltered in β-alanine-treated cells (Fig 4b). Lastly, we measured the protein expression of phospho-5’ AMP activated protein kinase (p-AMPK), widely accepted as the central regulator of cellular energetics; however, p-AMPK expression was unaltered by β-alanine treatment (Fig 4c).

## DISCUSSION

β-alanine has become a popular constituent in many commercially available sports supplements sold to improve athletic performance and prolong exercise duration^2, 3, 18^. To date, the mechanism by which these benefits are afforded has been attributed to increased carnosine biosynthesis leading to improved skeletal muscle buffering capacity^14, 18, 23^. However, this work characterized the influence of β-alanine on mitochondrial responses in skeletal muscle. We observed modest but consistent induction of several markers of mitochondrial biosynthesis and mitochondrial content in β-alanine-treated cells, suggesting β-alanine may improve skeletal muscle oxidative capacity. Specifically, our findings demonstrate that β-alanine’s effect on oxidative gene expression occurs in part through elevations in PPARβ/δ expression leading to increased mitochondrial content. Our findings further support this hypothesis through the observation of increased oxygen consumption in β-alanine treated cells compared to control; an effect abolished by concurrent PPARβ/δ inhibition. These findings are encouraging given that elevated muscle PGC-1α and PPARβ/δ expression are associated with improved fatty acid oxidation, glucose uptake/disposal, and reduced incidence of metabolic disease^1, 7, 20, 28, 29^. β-alanine treatment also promoted increased GLUT4 expression through MEF-2 induction, an adaptation which may prove beneficial for both athletics and metabolic disease^9, 17^, however β-alanine did not alter glucose uptake. Interestingly, despite β-alanine induction of PGC-1α RNA expression, we did not observe elevated PGC-1α protein expression. Given that β-alanine treatment led to enhanced expression of MEF-2 (a downstream PGC-1α target) with subsequent increases in GLUT4 content, β-alanine may promote PGC-1α expression (an observation which may have been missed at the tested time point). Additionally, it has previously been shown that PPARδ agonists can rescue suppressed TFAM expression independent of PGC-1α induction, suggesting PPARδ induction may be sufficient to independently promote mitochondrial biogenesis through TFAM up-regulation^8^. Moreover, the induction of PPARβ/δ suggests that β-alanine may promote lipid oxidation, observations which were partially verified by elevated CPT1b expression.

In addition to characterizing the effects of β-alanine on indicators of myotube metabolism, our findings also add an interesting finding regarding carnosine biosynthesis (specifically Carns1 expression). Because of limited carnosine bioavailability in the gut, supplementation with β-alanine tends to enhance muscle carnosine to a greater extent than carnosine supplementation through the Carns1 mediated reaction^6^. Everaert et al. measured the gene expression of multiple targets associated with carnosine synthesis and transport and reported no change in Carns1 mRNA expression in NMRI mice supplemented with 1.8% β-alanine drinking water for 8 weeks^6^. To our surprise, β-alanine treatment did not alter Carns1 content^14, 18, 23^. Despite numerous reports investigating the influence of β-alanine supplementation on muscle carnosine content, we were unable to locate a report documenting the effects of β-alanine supplementation on muscle Carns1 protein expression. Although speculative, it is conceivable that Carns1 increased carnosine synthesis by increased interactions with more available substrate in the β-alanine-treated cells (independent of expressional upregulation). The report by Everaert et al. also demonstrated a negative regulatory effect of androgens on muscle carnosine content^6^, suggesting a complex regulatory process overseeing cellular carnosine homeostasis beyond substrate availability (dynamics not investigated in our experiments).

β-alanine appears to stimulate the expression of several regulators of oxidative metabolism, mitochondrial biosynthesis, and possibly glucose uptake in cultured myocytes (observations which require in vivo verification). Details of the mechanism(s) by which β-alanine promote increased expression of PPARβ/δ remain unclear warranting further investigations into the exact mechanism(s) of action and physiological relevance of these observations.
